# Improving Stereotaxic Neurosurgery Techniques and Procedures Greatly Reduces the Number of Rats Used per Experimental Group—A Practice Report

**DOI:** 10.3390/ani11092662

**Published:** 2021-09-10

**Authors:** Barbara Ferry, Damien Gervasoni

**Affiliations:** 1 Centre of Research in Neuroscience Lyon CNRS UMR 5292-INSERM U 1028-Université Claude Bernard Lyon 1, Université de Lyon, 69675 Lyon, France; damien.gervasoni@univ-lyon1.fr; 2 Centre Hospitalier Le Vinatier—Bâtiment 462—Neurocampus, 95 Boulevard Pinel, 69675 Bron, France

**Keywords:** stereotaxic neurosurgery, refinement, reduction, 3R, well-being, rat model

## Abstract

**Simple Summary:**

Stereotaxic surgery techniques are commonly used today in research laboratories by a range of students, technicians, and researchers. Over the past twenty years, technical and scientific progress has been made in neurosurgery to meet the evolving requirements imposed by international legislation, and to promote the implementation of 3R rules. These improvements were motivated by a greater awareness of animal welfare and the necessary effort in the reduction of the number of animals used in experiments. The data presented in the present study show that technical and methodological improvements brought to our surgical procedures from 1992 resulted in reproducible stereotaxic neurosurgeries and in a significant reduction in experimental errors and animal morbidity. The effects of these improvements include a decrease in the final number of animals used in our experiments as well as better management of pain during and after surgery and the use of appropriate aseptic techniques. Correct stereotaxic surgical approaches are precisely described throughout the text.

**Abstract:**

Techniques of stereotaxic surgery are commonly used in research laboratories by a range of students, technicians, and researchers. To meet the evolving requirements imposed by international legislation, and to promote the implementation of 3R rules (replacement, reduction, and refinement) by reducing experimental error, animal morbidity, and mortality, it is essential that standard operating procedures and proper conduct following such complex surgeries be precisely described and respected. The present report shows how refinements of our own neurosurgical techniques over decades, have significantly reduced the number of animals (rats) used in experiments and improved the animals’ well-being during the post-surgical recovery period. The current pre-, per-, and post-surgical procedures used in our laboratory are detailed. We describe the practical aspects of stereotaxic neurosurgery that have been refined in our laboratory since 1992 and that cover various areas including appropriate anesthesia and pain management during and after surgery, methods to determine the stereotaxic coordinates, and the best approach to the target brain structure. The application of these optimal surgical methods that combine reliable and reproducible results with an acute awareness of ethics and animal welfare leads to a significant reduction in the number of animals included in experimental research in accordance with ethical and regulatory rules as required by the European Directive on laboratory animal welfare.

## 1. Introduction

Stereotaxic neurosurgery is used in combination with other diverse techniques necessary for addressing the specific objectives of a proposed neuroscience study. These techniques include in vivo experimental procedures for activating or inactivating brain structures or transmitter systems, permanent selective lesioning, functional neuroanatomy using tracers, and acute or chronic measurements or recordings. With the Directive 2010/63/EU for the protection of laboratory animals 2010 [1] and in line with “The Principles of Humane Experimental Technique” [2], Europe has reinforced the implementation of the 3Rs principle (replacement, reduction, and refinement) on the use of animals for experimental and other scientific purposes. As in the preceding Directive (86/609/EEC), appropriate formation and training are required for all those engaged in the use of stereotaxic neurosurgery in rodents for scientific purposes. A high standard level of skill in stereotaxic neurosurgery implies a good knowledge in various areas of expertise but also a great awareness of ethics and animal welfare.

While legal frameworks applicable to animal research have considerably evolved since the early 1980s, animal care and use guidelines and recommendations have also been provided and regularly updated by expert groups or entities (e.g., National Research Council 2011) [3], Canadian Council on Animal Care 2013 [4], Federation for Laboratory Animal Science Associations, and animal welfare bodies, see Guillén and Steckler for review [5]). Overall, in conjunction with regulatory incentives these helpful recommendations and advice have promoted better practices and improved the day-to-day follow-up and care of laboratory animals. Additionally, with the general public being more and more informed, animal researchers have also become increasingly aware of societal expectations on laboratory animal welfare. Compliance with the 3R principle, implementation of humane endpoints, and higher scrutiny in pain recognition and management have now become essential requirements.

Animals, especially rodents, are widely used in neuroscience research in order to study normal and abnormal brain functions [6]. Since 1992, our research has focused on the role of the central structures involved in learning and memory. In particular our experiments have addressed the involvement of several temporal lobe structures in the successive memory phases underlying associative learning tasks in the rat model. For this purpose, we have used different techniques aimed at testing the effect of local activation or inactivation of target structures on these processes. Such an approach involves the stereotaxic placement of guide cannulas in adult rats, that allow intracerebral injections (and collection) of fluids.

The present work describes the techniques based on common standard procedures of stereotaxic surgery, during which material can be inserted into the brain in order to microinfuse drugs in animals performing a task. Based on the data published or collected in laboratory notebooks, reports, and theses from Masters and PhD-students under our supervision, we provide a detailed report on the changes that were introduced over time in our experimental procedures. The reasons or causes that motivated the improvement made to our procedures throughout our research experience were based on research evidence. Systematic post-mortem observations of lesion or cannula placement and implementation of end-point assessment sheets, precisely highlighting the reasons why some animals were discarded from the final experimental groups, made it possible to target the areas in which techniques needed to be improved in order to reduce the number of animals discarded from the final experimental groups. In particular, this analysis showed that progress could be made in terms of asepsis and postoperative recovery. The lack of precision in reaching the target structure motivated the use of pilot surgery in which animals that had already been used in an experiment were reused under anesthesia in non-survival surgeries to improve the accuracy of the coordinates of the approach to the target structure. Finally, the growing literature on the benefits of new methods in analgesia, anesthesia, and asepsis also advanced our methods and refinements.

The analysis of the proportion of animals that were finally excluded from the experimental groups throughout our research clearly showed a positive impact of all these changes on the wellbeing of the animals, the reduction in the number of animals used and the quality of the experimental data. The description of the general methodology includes the chronological list of the refinements (modification, addition, or abandonment of a component of the overall procedure) that have been introduced in stereotaxic procedures during the period that covers our research activity from 1992 to 2018.

## 2. Materials and Methods

### 2.1. Implementation of Aseptic Techniques

#### 2.1.1. Environment and Materials

Aseptic techniques have received special attention since we started our experiments in 1992 and sterilization of the surgical equipment was a first step implemented in our procedures. To this end, surgical tools (surgical drapes, gowns, compresses, cannulas, electrodes, micrometer screws, obturators, tools used for drilling the skull bone, scalpels, retractors, scissors, and needle holders) were sterilized by placing them for 30 min at 170 °C.

The ear and incisor bars, the hand piece of the dental drill, and the stereotaxic frame were simply cleaned with disinfectant wipes.

From 1994 on, a change was introduced in the procedure and the cannulas were no longer sterilized by heat. Instead, they were put in a bath of hexamidine solution and the inside was systematically infused by a solution of hexamidine and rinsed with a sterile NaCl solution.

From 2005, we implemented, from the beginning to the end of surgery, a sequence of steps under the go-forward principle in order to limit contact between soiled and sterile instruments or materials and therefore to keep a high level of asepsis. The organization of space was included in this process with the delineation of two distinct areas, one called a “dirty” area for the preparation of the animal (see below), and the other, a “clean” zone for the surgery.

#### 2.1.2. Preparation of the Surgeon

From 2005 on, an assistant was always present and helped the experimenter to conform with the go-forward principle and to prepare the surgical set-up. After a thorough surgical handwashing, the surgeon is helped for gowning and gloving, with a sterilized gown and mask as well as sterile gloves prepared in advance. Once the surgeon is equipped, the assistant places the instrument box on a sterile drape next to the stereotaxic frame.

#### 2.1.3. Preparation of the Animal

A clinical examination is made to ensure that each animal has a good health status before proceeding. Importantly, none of the rats is subject to food restriction before surgery. The animals’ weight is carefully measured for the adjustment of anesthesia dosage and this is kept as reference for the post-surgical follow-up. An animal is first anesthetized and prepared (surgical shearing) in the “dirty” area. Second, the paws and tail of the animal are gently cleaned with an iodine or hexamidine scrub solution. The animal is then brought by the assistant to the “clean” zone. From 1994 on, a thermostatically controlled heating blanket with a rectal probe has been used to ensure an optimal control of internal body temperature. At this point, the surgeon takes charge, checks the depth of anesthesia and installs the animal into the stereotaxic frame by fixing its head between ear- and nose-bars. Blunt tip ear bars are used and care is taken for their insertion: first, the observation of a blink of the eyelids ensures an accurate positioning at the entrance of the external auditory canal and second, the scale on the bars is systematically used as an index of their progression and position. An ophthalmic ointment is applied on the eyes to protect the cornea from desiccation. The top of the animal’s head is then scrubbed with an iodine foaming solution (Vetedine Scrub^®^, Vetoquinol, Magny-Vernois, France), rinsed with sterile water, and disinfected with iodine solution (Vetedine Solution^®^, Vetoquinol, Magny-Vernois, France). Alternatively, chlorhexidine-based soap and solution (Hibitane^®^, Intervet SA, Beaucouzé, France) are used. The surgeon then lets the antiseptic solution dry out and checks the position of the head.

### 2.2. Anesthesia and Presurgical Analgesia

From 1992 to 1999, anesthesia was induced by an intraperitoneal (i.p.) injection of 5 mg/kg diazepam followed by 100 mg/kg ketamine [7,8]. From 1999 to 2006, anesthesia induced by i.p. injection of sodium pentobarbital (50 mg/kg) was supplemented with atropine sulfate (0.4 mg/kg, i.p.) to suppress salivation and bronchial secretions [9,10,11,12,13,14]. From 2006, anesthesia has been induced by i.p. injection of a mixture of 3/4 part of ketamine (Imalgene^®^, Boerhinger, Ingelheim, France; 100 mg/kg) and ¼ part of the alpha-2 agonist xylazine hydrochloride (Rompun^®^, Elanco France, La Garenne Colombes, France; 10 mg/kg) [15,16,17,18,19,20,21,22]. Anesthesia is maintained by reinjections of a half- or quarter-dose after an initial phase of 30 to 45 min.

From 1992, the animals received a subcutaneous injection of lidocaine (Xylocaine^®^, Aguettant, Lyon, France) at the incision site once deeply anesthetized (suspension of consciousness, loss of tail- or toe-pinch withdrawal reflex, pain sensitivity, and mobility [23,24]. From 2006, the animals also received a systemic analgesic through a subcutaneous injection of an analgesic dose of the mu opioid receptor agonists butorphanol (Dolorex^®^, Merck, New York, USA) 0.4 mg/kg and later on buprenorphine (Buprecare©, Animal Care, York, UK) 0.05 mg/kg in order to reduce pain during surgery [24,25].

### 2.3. Stereotaxic Surgery Procedure

Incision of the skin and exposure of the cranial surface is made using a scalpel with a sterile blade and tissue retractors are placed on each side of the cranial area of interest. The bone surface is gently scraped with a periosteal raspatory or a bone scraper. Sterile cotton-tips and saline solution are used to remove bone debris and dry the skull surface.

The cannula holder, or injection needle is installed on the object carrier and its verticality relative to the coronal and median planes is checked. The coordinates of the bregma and lambda landmarks are measured in order to position the skull in a horizontal position (“flat-skull”). The dorso-ventral (DV) coordinates of both landmarks are checked and the inclination of the head is adjusted whenever the difference between both DV coordinates exceeds 0.1 mm. Sterile anchor screws (when needed; for most procedures two or three screws are used) are then inserted to the skull to strengthen the fixation of the implant. To avoid damage to the underlying pia, only short-length screws are used.

From 1995, the “flat-skull” position was verified once. From 2004, the flat position of the skull was checked twice before and after the insertion of the anchor screws.

Then, the ML and AP coordinates of the target structure are calculated relative to the appropriate reference (bregma or lambda) with support of a reference atlas and its updated versions [26,27,28]. Importantly, stereotaxic coordinates are determined by taking the topography of the cerebral vascular system to avoid vascular structures that could be a source of bleeding and irreversible damage at the time of insertion. For this purpose, anatomical representations of blood vessels and publications showing brain angiographies are used [29,30]. Vertical approaches are used as much as possible, and care is taken to avoid branches emerging from the anterior, the middle, or the posterior cerebral arteries. The needle/cannula is then positioned above the target and its tip used to determine the final DV coordinate of the target from the skull surface. The skull is then drilled, and the dura is hooked using the tip of a small sterile hypodermic needle, and incised in a circular motion.

From 1995, the verification of the vertical position of the holders was also checked after having positioned the carrier on the micromanipulator on the stereotaxic frame.

From 2004, the reference point for the calculation of the final DV coordinates was taken from the dura surface. Indeed, our observations showed marked differences in skull thickness as a function of age, occasionally resulting in shifts in the DV position of cannulas across animals when using to the bone surface as a reference. The cannula, or injection needle, was installed on the object carrier after the dura was resected. This step was repeated in the case of bilateral insertion.

Then, the cannula or injection needle is carefully lowered according to the DV coordinates, with a speed of about 1 millimeter per minute.

In 2012, we introduced a new holder with grooves aligned at fixed intervals to maintain two cannulas strictly in parallel for bilateral implantations. This tool allowed the simultaneous insertion of two parallel guide cannulas in a single move of the holder. From this period on, the axial position of the skull was also systematically verified before inserting the material.

When the material has been inserted correctly in the brain, the skull is dried and a self-curing adhesive resin (Super-Bond C&B^®^, Sun Medical Co., Moriyama-shi, Japan) is applied [31]. The guide cannula and anchor screws are then fixed solidly to the skull of the animal using dental acrylic cement (e.g., Unifast Trad^®^ powder and solvent, GC America Inc., Alsip, IL, USA). The cement is applied using the brush-dip technique which consists of forming a ball of powder/liquid at the tip of a brush, first by dipping the tip in the liquid and then touching the polymer powder. Compared to the bulk-mixing technique, this mode of application results in a high powder/liquid ratio that limits the thermal effect of polymerization, fastens the curing, and shortens the working time. The edges of the implant are made as smooth as possible. When the cement has completely dried, the object carrier is slowly raised leaving the guide cannula in place and visible on the skull. A sterilized obturator (stylet) is then inserted into the guide to avoid clogging during the post-operative recovery period. Depending upon the size of the skin incision and the volume of the implant, the wound is sutured at the end of the procedure. For this purpose, sterile suture material is used. Single stiches are done using resorbable suture wire on a 3/8 curved needle. At the end of the procedure, the wound is cleaned with a compress soaked with a 1:10 dilution of chlorhexidine in sterile water. Any trace of cement, hair, or blood clots or other debris that can interfere with wound healing is removed. Before the animal fully recovers from anesthesia, the nails of all paws are trimmed to minimize any impact of the animal’s normal scratching and grooming behavior on the healing process. The return of the palpebral reflex is checked using a cotton-tip applied on the eye’s corner.

From 1995, pilot studies were conducted with injections of dye at the target sites and immediate histological verification of the position of dye deposits to determine the correspondence between the theoretical coordinates and actual placement within the target area. Occasional discrepancies between actual and atlas coordinates were thus corrected [32].

From 2004, cannulas with reduced diameter were used (from 22 to 23 gauge).

### 2.4. Monitoring Vital Signs during Surgery

Toe- or tail-pinch withdrawal reflexes, breathing rhythm and amplitude, color of the mucous membranes, body temperature, and possible dehydration of the animal are checked throughout the surgery. This monitoring has been applied since 1992. From 1994, in addition to the visual control of the animal’s breathing and the color of its mucous membranes, internal temperature has been systematically monitored and maintained throughout the surgery.

### 2.5. Post-Surgical Procedure

At the end of the surgical procedure, the animal is temporarily placed in the recovery zone with an infrared spot above the cage. Importantly, the light is placed such that an animal can move away from the hot spot once having recovered its righting reflex. Post-operative care procedures such as manual feeding and facilitated access to drinking source (by lowering the drinking spout of the water bottle) are provided until an animal recovers its autonomy and a complete restoration of normal behavior. Each animal returns to the housing facilities only after complete awakening from anesthesia and is placed back in its home cage with its cage mates.

From 1992 to 1994 systematic i.p. injection of 5 mL of NaCl and heating of the animal at the end of surgery were used. From that period, animal facility staff were involved in daily post-surgical care and nursing. Individual follow-up sheets were filled for each animal and listed all the substances an animal had received pre- per-, and post-surgery. In some rare circumstances of severe complication, the involvement of the technical staff enabled rapid euthanasia according to predefined endpoints, thus avoiding unnecessary suffering. Also, from 1992 to 2000, a prophylactic antibiotic treatment (one injection of penicillin 0.12 MU/0.4 mL, i.m.) was used in order to complete our aseptic techniques [7,8,9,33].

From 2012, i.p. injections of 5 mL of glucose Ringer lactate 5% replaced the use of NaCl at the end of the surgery and all animals received a subcutaneous injection of a nonsteroidal anti-inflammatory drug (carprofen, Rimadyl^®^, Zoetis France, Malakoff, France; 12–24 h duration of action). An evaluation of signs of distress, pain, and discomfort (i.e., anorexia, weight loss, self-injurious behavior, altered state of consciousness, difficult breathing, abnormal color/appearance of the mucous membranes, abnormal neurological signs, and changes in locomotor function affecting drinking and food intake) was done daily and observations were written on the individual’s follow-up sheets [9,33]. Post-operative pain was scrutinized with a specific score sheet that includes appearance and overt behavioral signs, the reaction to the manipulation of the operative zone and intensity of this reaction, and a subjective global score. Anti-inflammatory treatment was maintained as long as the animal showed signs of distress by additional carprofen injections. Analysis of the scoring sheets revealed that the duration of the treatment rarely exceeded 48 h. Contemporaneous records of each animal served to prepare a final report on animal use, and to determine the actual level of pain and distress of the procedures. With the improvement of the aseptic conditions, and in accordance with the legislation that became much stricter with the antibiotic use in animal research due to the development of resistance to particular germs observed in certain breeding farms. Therefore, antibiotic prophylaxis was discontinued in 2000.

### 2.6. Pharmacological Experiments

Animals were allowed to completely recover from the surgery for at least two weeks before performing behavioral and pharmacological studies. During this period, the experimenter visited the animals once daily, and checked the integrity of the implant and wound, as well as the operability of the guide cannula. For this purpose, each animal was carefully handled and restrained by hand while the experimenter checked the presence of the stylet and the absence of resistance when sliding it up and down. This daily handling contributed to the habituation of the animals to the experimental conditions. The injected drugs were always freshly prepared on the day of the experiment using aseptic techniques and frozen aliquots of sterile medium (sodium chloride 0.9% or artificial cerebrospinal fluid solution). In addition, the inside of the tubing was infused with a 70% alcoholic solution before each microinfusion. From 1994 on, the animal care staff was involved in the follow-up of the animals and would inform the experimenter whenever an abnormal or unexpected situation occurred.

### 2.7. Post-Mortem Examination of Animals

After completion of behavioral and pharmacological testing, each rat was euthanized for brain removal and histological examination of the injection sites. Euthanasia was performed with an overdose of anesthetic.

### 2.8. Statistics

All analyses were performed with Prism (GraphPad Software Inc. San Diego, CA, USA). For normalization purpose, a ratio of the “total number of animals discarded from the statistical analyses/total number of animals used in experimental research in our group from 1992 to 2018” was calculated and analyzed. The Agostino Pearson’s analysis was used to test the normality of the distribution and this was followed by Pearson’s parametric correlation test.

## 3. Results and Discussion

Data were obtained from a total of 113 studies and reports in our research group from 1992 to 2018. A total of 3228 animals, weighing 250–300 g at surgical period, were involved in the present study (*n* = 348 Wistar; *n* = 1880 Long–Evans, and *n* = 1000 Sprague-Dawley strains). The number of animals finally discarded in each study is compiled and presented as a proportion for normalization and inter-study comparison purposes. No effect of strain was ever observed during our experiments, whatever the refinement considered.

**Figure 1:**
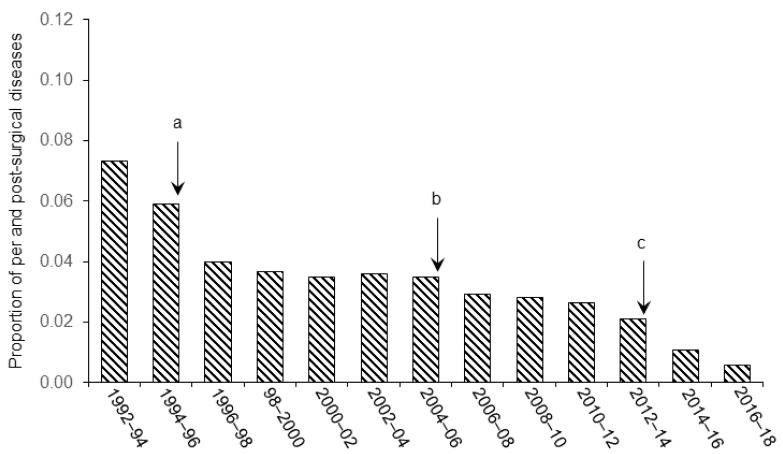
Effect of refinement in anesthesia, pain management, and rehydration on animal post-surgical recovery. Letters indicate the chronology of various improvements introduced in the surgical procedure. (**a**) From 1994 to 1996, internal temperature has been monitored and maintained. Systematic i.p. injection of 5 mL of NaCl and heating of the animal at the end of surgery have been used. Involvement of animal facilities staff in pain control methods including daily post-surgical care and nursing. Individual follow-up sheet which lists all the substances the animal received during the different periods covering the surgery (pre-, per-, and post-surgery) and daily evaluation of signs of pain and distress is completed for each animal. (**b**) From 2004 to 2006, diazepam/xylazine or sodium pentobarbital anesthetics have been gradually replaced by the association of ketamine and xylazine. (**c**) From 2012, systematic i.p. injections of 5 mL of glucose Ringer lactate 5% and a subcutaneous injection of an anti-inflammatory have been given at the end of the surgery in order to control pain.

**Figure 2:**
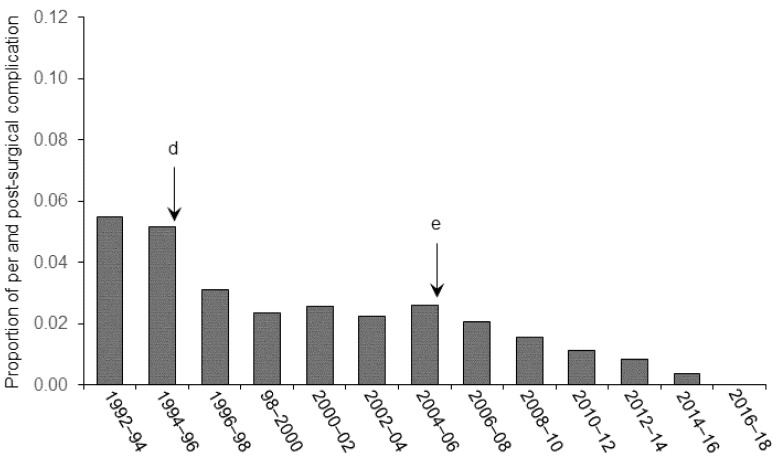
Effect of refinement in the surgical and microinjection procedures on septic complications. Letters indicate the chronology of various improvements introduced in the procedures. (**d**) From 1994, the inside of the cannulas was systematically sterilized. In addition, during the microinfusion procedure, the inside of the tubing, the injection needles, and the injected drugs were prepared in careful aseptic conditions. (**e**) From 2005, we implemented an asepsis chain following a “go-forward” workflow.

**Figure 3:**
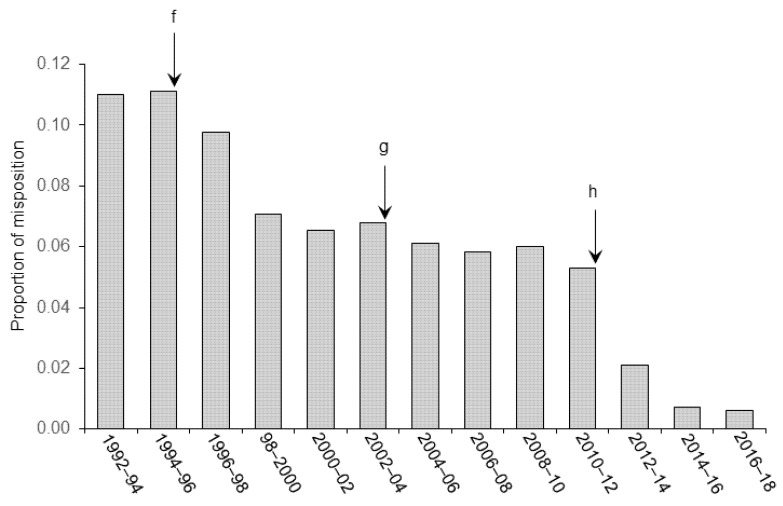
Effect of refinement in the stereotaxic procedure on the accuracy of placement of the implanted item(s). Letters indicate the chronology of the various improvements introduced in our procedure. (**f**) From 1995, verification of the flat-skull position before each surgical session. Verification of the vertical position of the holders. Introduction of pilot studies and immediate histological verification of implantation site to check the correspondence between the theoretical coordinates and actual placement within the target area. (**g**) From 2004, reduction of the cannula diameter. Final DV coordinate of the target structure is calculated from the dura and systematic verification of the flat position of the skull after screw position and on each animal. (**h**) From 2012, development of a fixed spaced slide for cannulas (bilateral implantation) and systematic verification of the axial position of the skull. Most of the data presented on the graph corresponds to the basolateral and central amygdala, and medial and lateral entorhinal cortex targeted structures.

The proportion of animals that were discarded from the final statistical analyses are spread relative to the category of improvement brought in our experimental procedure. Figure 1 shows the effect of refinements in anesthesia and control of vital signs on the general recovery of the animals (clinical signs of post-surgical pain and distress), Figure 2 illustrates the effect of improvement of aseptic techniques on the number of infectious complications (internal and external), and Figure 3 shows the effects of stereotaxic methodology improvement on the number of animals discarded because of cannula misplacement.

As illustrated in the figures, we observed significant decreases in the number of animals excluded from final analyses following the implementation of technical (in stereotaxic pre-, per-, and post-surgery) and welfare (pain and distress management, anesthesia) refinements in our procedures from 1992. Statistical analyses confirmed these observation and Pearson rank correlation revealed a significant relationship between the proportion of animals discarded from the analyses and the refinement in the different techniques throughout the successive periods of research (r = −0.93, *p* < 0.001 for Figure 1; r = −0.94, *p* < 0.001 for Figure 2 and r = −0.95, *p* < 0.001 for Figure 3). These refinements concern different areas of expertise that are listed below.

### 3.1. Pain and Distress

Figure 1 shows how technical improvements in our anesthesia and pain management procedures positively impacted post-surgical welfare of the animals. While the priority in 1992 was to monitor the proper depth of anesthesia during the operation by checking the various reflexes, a gradual increase in the attention to general homeostasis, body temperature, and proper hydration maintenance of the animal encouraged us to complete our surgical procedure. As shown in Figure 1 (event a), improvements brought to the monitoring technique from 1994 led to a substantial decrease in the post-surgical mortality of the animals. From 2004 (event b), replacement of diazepam/xylazine or sodium pentobarbital by the association of ketamine and xylazine improved the overall quality of surgical anesthesia, by reducing the time of induction and nociception and increasing myorelaxation [34]. Moreover, from 2012, other refinements such as post-operative management of pain by administration of a NSAID at the end of the procedure, involvement of the animal facility staff in the follow-up of health and surveillance of clinical signs, and modification of the rehydration procedure resulted in a reduction of the mortality rate. Postoperative scoring sheets facilitated the implementation of endpoints, for instance when an animal showed persistent pain 48 h after surgery despite the use of analgesics.

The welfare of animals included in an experiments is essential to ensure reliable and reproducible results. From their arrival in the laboratory facilities, animals were kept under constant supervision in order to detect signs of pain and distress as early as possible. Recognition of these signs can sometimes be difficult especially in prey animals, but helpful information can be found in the literature [25,35,36]. From 1994, animal caretakers were trained to evaluate post-operative signs of distress and involved in the daily follow-up of animals. This involvement of the animal facility staff improved the level of animal care and nursing.

Minimizing pain and discomfort during (pre- and per-surgery) and after a surgery relies strongly on the choice of sedation, anesthetic and analgesic compounds, and an appropriate rehydration procedure when needed, but also on the monitoring procedure. Depth of anesthesia, and vital signs such as respiratory rhythm and amplitude, should frequently be checked throughout surgery in order to react rapidly. General anesthesia causes a dose-dependent impairment of thermoregulation, a depression of cardiovascular and respiratory systems [37,38], a lowering of metabolic activity, and a peripheral vasodilation that can lead in a matter of minutes to a drop in body temperature and a fatal hypothermia under 32 °C. Moreover, prolonged hypothermia during a surgery can lead to difficulties in awaking, discomfort, and complications from poor blood perfusion, inadvertent coagulation, and ischemia. Conversely, hyperthermia might lead to death. Since a faulty heating blanket or probe can cause severe harm, appropriate checking and maintenance of heating devices is recommended when preparing the surgery and attention should be paid to their correct functioning throughout the procedure. From a general point of view, a good monitoring procedure can prevent complications occurring during surgery.

### 3.2. Surgical Asepsis

Surgical asepsis refers to the absence of infectious microorganisms during an invasive procedure. Such organisms are naturally present in the environment, in animals and humans, and on all foreign material that can be introduced in a living tissue (biofilm). Aseptic techniques are thus essential to prevent infection of the surgical site and post-surgical complications and to minimize the risk of mortality.

Figure 2 shows the effect of asepsis-related refinements on post-surgical septic complications observed post-mortem. The implementation of the daily evaluation and observation of signs of distress procedures in 1994 enabled us to intervene and cure an external infection at the surgical site so that none reached any of the endpoint’s criteria leading to euthanasia. Animals that were discarded from the final analysis corresponded exclusively to those showing a post-mortem gliosis resulting from a tissue inflammation. As shown in the figure, a first noticeable decrease in the number of animals discarded from the analyses (in which the gliosis reaction covered the site of microinfusion) was observed in the period 1994–1996 (event d). Fewer parenchymal over-reactions were observed post-mortem when surgical tools and the material such as cannulas, anchoring screws, stylets, and cannula holders were chemically decontaminated in a bath of antiseptic solution. Special attention was paid to the decontamination of the insides of the cannulas. Stylets that were replaced in the cannulas throughout the experiment were always decontaminated with 70% alcoholic solution before being re-inserted. The second important event that influenced the number of animals discarded from the final experimental groups after the 2004–2006 period (event e) corresponded to the enforcement of the “go-forward” process. With the clear spatial delineation of three zones (i.e., “dirty”, “intermediate”, and “clean”), we were able to implement a “go-forward” workflow whatever the type of manipulation, i.e., the surgery itself, single microinfusion, or stylet replacement. As shown in the figure, all these refinements led to a sustained reduction in the number of animals used in the analyses so that today there are no more animals excluded from experimental groups due to a contamination event.

### 3.3. Stereoaxic Coordinates and Angle of Approach during Surgery

When determining stereotaxic coordinates of a target structure one should consider its anatomical and functional characteristics, its spatial coordinates as given by the atlas, the organization of the cerebral vascular system, and the age or state of development of the animal. In order to ensure a high precision and reproducibility of stereotaxic surgeries, these should all inform the implantation of materials such as cannulas, electrodes, or microdialysis probes.

In this regard, from 1992, a series of improvements have been brought to our stereotaxic calculation of the brain target coordinates by a systematic verification of the adequacy between the theoretical position of the target structure found in the atlases [26,27,28], and its actual position in our animal model [32]. In addition, the cerebrovascular anatomy, especially the arrangement of the leptomeningeal vessels has been taken into account for the calculation of the angle of approach in order to avoid critical danger of rupturing important vessels such as the transverse sinus during material insertion [39,40,41].

As shown in Figure 3, the first event observed after the 1994–1996 period corresponded to a refinement in the method to verify the angle of insertion of the cannula into the brain. From that period, the flat position of the skull was verified before each session of surgery. To do this, the correspondence between the DV (±0.1 mm) coordinates measured at the lambda and bregma reference points was verified before inserting anchoring screws. From that period, we also systematically verified the vertical alignment of the cannula holders. Both operations significantly contributed to improve the repeatability in the intracerebral A/P placement of the cannula. In addition, pilot non-survival surgeries and immediate histological verification of implantation site were conducted during that period on animals previously used in other experiments. This helped to adjust the stereotaxic coordinates indicated in the Paxinos and Watson atlas in Wistar rats (1) to those found in other type of rats (Sprague Dawley, Long–Evans) and (2) to those found in rats of a different weight.

The second important improvement was observed during the 2002–2004 period when cannulas of a smaller diameter were introduced (from 23 to 24 gauge); less invasive, thinner cannulas have a better tissue tolerance and allow a more precise positioning into the brain. Also, from that period, due to the imprecision observed in some cases between the theoretical DV coordinate of the target structure and the position of the canula observed post-mortem (i.e., convex shape of the cranial bone), the final D/V coordinate of the target structure was calculated from the dura. In addition, from 2002, the flat position of the skull was verified before and after the screw anchoring.

The third significant progress in the method appeared in the 2010–12 period and resulted from the development of custom-made holders maintaining a given distance and a strict parallelism between cannulas for bilateral implantations in symmetric areas. Such holders were manufactured with parallel groves to fit exactly the M/L distance between each structure targeted. Also, the M/L axial flat skull position was systematically verified in order to ensure perfect vertical position and correct M/L position of the cannulas. To this aim, the D/V coordinates of the point above the target structure was bilaterally checked for correspondence.

As shown in the figure, these refinements led to a steady decline of the number of animals discarded from the analyses due to misplacement of lesion or injection sites. Our method has improved in accuracy and reproducibility so that only 1/166 subject was excluded from the final analysis in the final period compared to 12/109 during the first period.

### 3.4. Further Refinement Perspectives

The refinements introduced in our research are somewhat diverse and might not apply to all stereotaxic surgeries. Stereotaxic surgeries can indeed be used in a variety of research projects; while general recommendations can apply, such as the systematic use of aseptic techniques in survival procedures, others need to be considered on a case-by-case basis. This seems particularly true when considering the nature of anesthetic and the analgesic and other drugs to administer to an animal. Dosage and administration route are obvious parameters, but others such as half-life and potential side-effects also deserve attention, especially in neuroscience research. Some drugs acting on the central nervous system can have delayed effects that can impact the animal’s behavior and well-being. For example, compounds like buprenorphine [25] or fentanyl offer effective pain relief properties but can also cause nausea [42] and a decrease of intestinal motility [43,44]. Some anesthesia methods can cause excessive salivation and bronchial secretion. To minimize such effects, experimenters can consider the use of atropine sulfate. Because of its central parasympathomimetic effect, especially at low dose, atropine sulfate can also induce bradycardia instead of tachycardia. Experimenters can alternatively consider glycopyrrolate (Robinul^®^) that does not cross the blood–brain barrier or choose another anesthesia method that does not cause excessive secretions. The choice of a drug should also take into account the potential delayed effect, such as anxiety signs, that may need a specific test to be objectivated.

Regarding anesthesia in our own research, we observed a significant decrease in mortality following the refinement in the method. As an alternative to fixed anesthesia, we have introduced inhalational anesthesia, since it has been shown to be safer and more effective than parenteral anesthetics [45,46]. Isoflurane, the most-used compound [47] has a rapid induction and awakening due to its low solubility [48]. It provokes little or no salivation or tracheobronchial secretions. By adjusting the vaporizer, the level of anesthesia can be rapidly adapted. Before surgery, the animal is placed in the “induction box” where it receives a mix of 2 to 3% isoflurane. Once deeply anesthetized, the animal can be transferred in the stereotaxic frame where the nose bar is equipped with a mask through which a mix of 0.25 to 2% of isoflurane is applied for maintenance. Moreover, a supplement of oxygen should be used during surgery to avoid hypoxia [49]. In order to control this, several devices now exist for monitoring blood gases (oximetry, capnography) and arterial pressure in rodents. Although not widely considered as being necessary for operatory procedures of relatively short duration, they will be tested in the near future for use in our laboratory during long surgeries.

Because of its poor analgesic properties and its rapid elimination, switching to isoflurane anesthesia prompted us to reconsider our analgesia protocols. Since pain can be much more difficult to control once the animal has become conscious of it, we systematically use local anesthetic drugs (lidocaine, bupivacaine), and opioids (butorphanol, buprenorphine) for surgery and a post-operative 48 h treatment with NSAIDs (carprofen) to provide good analgesic levels and comfort for the animals.

Any significant change in a procedure should therefore be thoroughly considered before being implemented. A cost/benefit approach might be appropriate in this matter. Such methodology implies a careful assessment of many parameters, not only during the surgery itself, but also during the immediate and long-term periods that follow. These parameters can strongly differ between research studies. When targeting a brain area involved in motor control, the follow-up of an animal’s well-being should include signs of motor deficits or handicap. Such signs can be extremely subtle, or even somehow compensated, but they should always be anticipated. Animal caretakers are the first-line actors, because of their presence in animal facilities, but refining a complete procedure will also benefit from close interactions between experimenters, the animal welfare body involved in the follow-up of projects, and the designated veterinarian. If required by the ethical oversight body or institutional animal care and use committee, a retrospective assessment can also provide guidance and suggestions for future improvements.

Although the proportion of animals excluded from our experiment today has reached a low level, we continue our efforts to further improve our procedure and the welfare of the animals without forgetting the well-being of the experimenter and facility staff, bearing in mind that good working conditions (physical and mental) will contribute to high-quality care to the animals and limit compassion fatigue. For this purpose, we regularly update and enrich our training program for experimenters and caretakers in our animal facility on stereotaxic surgery, nursing skills, and ethical requirements. Such training includes surgery planning, stereotaxic apparatus handling, determination of stereotaxic origin, coordinate calculations, and device placement. We also consider implementing technical innovations such as digital stereotaxic rulers and motorized arms which can contribute to improve targeting accuracy [49,50].

## 4. Conclusions

Over the past twenty years, technical and scientific progress has been made in neurosurgery to meet higher ethical standards. These improvements of pre- per-, and post-surgical approaches were motivated by a greater awareness of animal welfare and the necessity to reduce the number of animals used in experiments. We identified three major domains where improvements allowed us to achieve reliable and reproducible stereotaxic neurosurgeries with a lower number of subject rejections. These domains include (1) management of pain during and after surgery, (2) appropriate aseptic techniques, and (3) correct determination of stereotaxic coordinates and surgical approaches.

The data presented in the present study show that refinements made in our procedures from 1992 to 2018 resulted in a significant reduction in the number of animals used in experimental research through continuous decrease in experimental errors and animal morbidity over time. Resulting from the refinement brought to our procedures, the implementation and completion of postoperative scoring grids revealed a decrease in the number of post-surgical reinjections of NDAIS, a decrease in the use of euthanasia after passing the endpoints, and a faster recovery of normal weight. These observations suggest the refinements probably impacted the well-being and the survival of the animals. These technical and methodological improvements were guided by growing ethical and animal welfare considerations which prompted us to evaluate and implement new practices and technologies found in the literature and throughout numerous exchanges within the neuroscience community.

## Data Availability

Data are available on request to the corresponding author.
